# Construction of EMT related prognostic signature for kidney renal clear cell carcinoma, through integrating bulk and single-cell gene expression profiles

**DOI:** 10.3389/fphar.2023.1302142

**Published:** 2023-11-15

**Authors:** Qi Huang, Feiyu Li, Li Liu, Rui Xu, Tao Yang, Xiaoyun Ma, Hongmei Zhang, Yan Zhou, Yongxiang Shao, Qiaofeng Wang, Haifeng Xi, Yancai Ding

**Affiliations:** ^1^ Institute of Medical Sciences, General Hospital of Ningxia Medical University, Yinchuan, China; ^2^ Department of Urology, The 942 Hospital of PLA, Yinchuan, China; ^3^ Department of Laser, General Hospital of Ningxia Medical University, Yinchuan, China

**Keywords:** kidney renal clear cell carcinoma, epithelial-mesenchymal transition (EMT), single cell analysis, prognostic signature, LGALS1

## Abstract

**Introduction:** Kidney renal clear cell carcinoma (KIRC), as a main type of malignant kidney cancers, has a poor prognosis. Epithelial-mesenchymal transformation (EMT) exerts indispensable role in tumor progression and metastasis, including in KIRC. This study aimed to mine more EMT related details and build prognostic signature for KIRC.

**Methods:** The KIRC scRNA-seq data and bulk data were downloaded from GEO and TCGA databases, respectively. The cell composition in KIRC was calculated using CIBERSORT. Univariate Cox regression analysis and LASSO Cox regression analysis were combined to determine the prognostic genes. Gene set variation analysis and cell-cell communication analysis were conducted to obtain more functional information. Additionally, functional analyses were conducted to determine the biological roles of si-LGALS1 *in vitro*.

**Results:** We totally identified 2,249 significant differentially expressed genes (DEGs) in KIRC samples, meanwhile a significant distinct expression pattern was found in KIRC, involving Epithelial Mesenchymal Transition pathway. Among all cell types, significantly higher proportion of epithelial cells were observed in KIRC, and 289 DEGs were identified in epithelial cells. After cross analysis of all DEGs and 970 EMT related genes, SPARC, TMSB10, LGALS1, and VEGFA were optimal to build prognostic model. Our EMT related showed good predictive performance in KIRC. Remarkably, si-LGALS1 could inhibit migration and invasion ability of KIRC cells, which might be involved in suppressing EMT process.

**Conclusion:** A novel powerful EMT related prognostic signature was built for KIRC patients, based on SPARC, TMSB10, LGALS1, and VEGFA. Of which, si-LGALS1 could inhibit migration and invasion ability of KIRC cells, which might be involved in suppressing EMT process.

## 1 Introduction

As the third main malignant genitourinary tumor, renal cell carcinoma (RCC) constitutes more than 90% of all malignant kidney cancers (Oto et al., 2020). It has been estimated that there are over 400,000 new RCC cases, leading to more than 170,000 deaths annually around the world (Bray et al., 2018). Kidney renal clear cell carcinoma (KIRC) has been the predominant histopathological subtype among all RCC, accounting for over 75% of all RCCs (Bokhari and Tiscornia-Wasserman, 2017) and over 85% of metastatic RCC cases (Ricketts et al., 2018). Currently, computerized tomography and histopathological analyses serve as the gold standards to diagnose KIRC (Zhang et al., 2021). Moreover, many novel treatment strategies for KIRC have been recommended during the past few years, such as single/combinatorial use of anti-PD-1 antibody (George et al., 2019). Unfortunately, some of KIRC patients have metastatic diseases at their first diagnosis, and the 5-year overall survival (OS) rate of metastatic KIRC patients is less than 10% (Mitchell et al., 2018; Liu et al., 2021a). The prognosis of KIRC patients is overall frustrated. It has been indicated that approximately 30% KIRC patients undergo recurrence or metastasis after nephrectomy (Xu et al., 2023a), which is a big obstacle in improving the prognosis. Although increasing studies have explored various diagnostic or prognostic biomarkers for KIRC (Wu et al., 2023), novel reliable markers are still urgently needed to optimize the early KIRC detection and clinical treatment strategies, in order to further improve the prognosis of KIRC patients.

Metastasis and recurrence are the predominant fatal causes for many malignant tumor patients, and KIRC is no exception. Epithelial-mesenchymal transformation (EMT), as a reversible cellular process, has been widely reported in many cancers regarding its indispensable role in tumor progression and metastasis (Cen et al., 2023), including renal cancer (Piva et al., 2016). There are totally three types of EMT process, of which type III has been regarded as an important process in tumorigenesis and tumor metastasis (Pastushenko and Blanpain, 2019; Bakir et al., 2020). During the EMT process, the cells gradually lose their epithelial phenotypes and change to mesenchymal cells, along with great migratory and even invasion ability obtaining (Roche, 2018; Feng et al., 2022). Zhong et al. have recently documented that LIMD2 is found to activate the ILK/Akt pathway in KIRC via inducing EMT process, thereby promoting the malignant progression and poor prognosis of KIRC (Zhong et al., 2022). Moreover, another study has constructed a prognostic model based on twelve EMT-related lncRNAs for KIRC, exhibiting good predictive performance (Xia et al., 2022). Additionally, in ovarian cancer, it has been indicated that SLFN5 is able to promote EMT, besides EMT and invasion movement could be significantly inhibited by SLFN5 silencing (Xu et al., 2023b). Collectively, accumulating studies have evidenced that EMT exerts tumor promoting role via multiple pathways and mechanisms (Kim et al., 2016; Wang et al., 2018). Hence, it is a promising direction to further focus on EMT related genes in KIRC via integrating single-cell RNA-sequencing (scRNA-seq) data and bulk RNA-sequencing data.

The genetic abnormalities and intratumoral heterogeneity of KIRC greatly affect the patients’ distant metastasis and drug resistance, thereby influencing prognosis (Wang et al., 2018). More recently, scRNA-seq has emerged as a powerful tool to clarify thousands of cells per tumor (Wang et al., 2018), which is conducive to understanding the intratumoral heterogeneity of various cancers. Accordingly, we herein jointly analyzed the KIRC scRNA-seq data and bulk RNA-sequencing data, in order to build reliable EMT related signature for KIRC patients. Our data are promising to provide more reference information for better KIRC clinical treatment strategies.

## 2 Materials and methods

### 2.1 Data collection

Firstly, the TCGA-KIRC cohort was downloaded from The Cancer Genome Atlas (TCGA) database (https://tcga-data.nci.nih.gov/tcga/), using TCGAbiolinks package of R. There were 72 pairs of KIRC samples and adjacent normal samples in TCGA-KIRC cohort, and the detailed sample information were listed in Supplementary Table S1.

In addition, we also obtained three scRNA-seq datasets GSE178481, GSE156632, GSE159115 from Gene Expression Omnibus (GEO) database (http://www.ncbi.nlm.nih.gov/geo/). The scRNA-seq data of totally 32 pairs of samples (KIRC samples and adjacent normal samples) were obtained from 16 KIRC patients (Supplementary Table S2). The expression data of 101 KIRC samples in E-MTAB-1980 cohort (ArrayExpress) were also included in this study, and the detailed sample information were listed in Supplementary Table S3 (Wang et al., 2018).

### 2.2 scRNA-seq data analysis

The raw sequencing reads were aligned to the GRCh38 human reference genome. Feature-barcode matrices were then generated using the Cell Ranger with the standard pipeline. Then eligible cells were selected according to the following criteria: 1) gene numbers between 200 and 20,000; 2) unique molecular identifier (UMI) count >300; 3) mitochondrial gene percentage <40%. After screening the top 2,000 highly variable genes (HVGs) from the normalized matrix, the principal component analysis (PCA) was used for the dimensionality reduction. The clustering analysis was conducted using Louvain clustering algorithm from Seurat (Aran et al., 2019). The top 30 PCs were selected for uniform manifold approximation and projection (UMAP) to visualize the cell clustering results (Becht et al., 2018). Cell types were annotated by known cell markers, employing SingleR package (Aran et al., 2019).

For a systematic analysis of cell-cell interaction, we further performed the cellular communication analysis, utilizing Cellchat with default parameters (Jin et al., 2021).

### 2.3 Immune cell infiltration analysis

Next, the immune cell infiltration in KIRC was analyzed using CIBERSORT (Newman et al., 2015). The marker genes of different cell types in scRNA-seq data were obtained using scMappR (Sokolowski et al., 2021), which were taken as the input data of CIBERSORT. The profile generated from scMappR was then employed to conduct deconvolution analysis on bulk data in TCGA-KIRC cohort.

### 2.4 Survival analysis

Regarding the prognosis of different groups, survival and survminer R packages (https://CRAN.R-project.org/package=survminer) were used to estimate the overall survival (OS) basing on Kaplan-Meier method. The difference significance of OS among different groups was determined by log-rank test.

### 2.5 Functional enrichment analysis

The functional information was analyzed using gene set variation analysis (GSVA), employing MSigDB signature gene sets “Hallmark” (https://www.gseamsigdb.org/gsea/msigdb/index.jsp). The pathways with *p*-value < 0.05, logFC > 0.5 were considered statistically significant.

### 2.6 Cell culture and qRT-PCR analysis

The human embryonic kidney cell line HEK293T and renal cell carcinoma cell line 786-O were both cultured in Dulbecco’s modified Eagle medium (DMEM; Gibco, Grand Island, NY, United States) containing 1% penicillin/streptomycin and 10% fetal bovine serum (Gibco). The cells were placed in a humidified incubator maintained at 37°C with 5% CO_2_.

Total RNA was extracted from the cells using TRIzol Universal total RNA extraction reagent (Invitrogen, Carlsbad, CA, United States). The quality and concentration of the extracted RNA were assessed using an UV spectrophotometer. Once qualified, reverse transcription was done using Transcriptor First Strand cDNA Synthesis Kit (GenStar, Beijing, China). Subsequently, qPCR assay was conducted employing LightCycler 480 Fluorescence Quantitative System (Roche, Basel, Switzerland). The reference gene was β-actin, and the primer sequences were listed in Supplementary Table S4. The mRNA expression levels were calculated according to the 2^−ΔΔCT^ method (three repeats).

### 2.7 Cell transfection

LGALS1 knockdown was generated using small interfering RNAs (siRNAs). In addition, LGALS1 siRNA sequences were included in Supplementary Table S5. Briefly, cells were seeded at 50% confluence in a 6-well plate and infected with negative control (NC), and knockdown (si-LGALS1). All transfections were carried out with Lipofectamine 3000 (Invitrogen, Carlsbad, CA, United States).

### 2.8 Scratch assay

A total of 7×10^5^ cells were seeded in each well of a 6-well plate for 24 h. A line was drawn in the middle of the well with a 10 μL pipette tip. After washing with PBS twice, cells were cultured for 24 h in a 37°C incubator. Then, wounds were photographed by microscope at different time intervals. The distances of the wounds were measured by photoshop.

### 2.9 Migration and invasion assays

The migration and invasion capacities of si-control and si-LGALS1 cells were analyzed by polycarbonate membranes (8 μm pore) in 24-well transwell chambers (Coring, NY, United States). About 1×10^4^ cells in serum-free medium containing 0.1% BSA were added to the upper chamber. The medium supplemented with 0.1% BSA and EGF (50 ng/mL, MCE, NJ, United States) were added into the down chamber. After 24 h incubation, cells in the upper chamber were completely scraped and trans to the lower membrane. The polycarbonate membranes were fixed and stained with Giemsa solution (Solarbio, Beijing, China) and photographed by microscope.

For invasion assay, transwell chambers were coated with prediluted extracellular matrix (3 mg/mL, Merck, Darmstadt, Germany) for 1 h before adding cells on the upper chamber. The next steps were conducted similarly to the above transwell migration assay.

### 2.10 Statistical analysis

Wilcoxon’s rank-sum test was used to determine the difference significance between cancer samples/tumor cells and adjacent samples/normal cells. The univariate Cox regression analysis was performed using the survival package of R. Receiver operating characteristic (ROC) analysis and area under curve (AUC) calculation were conducted using survivalROC package. The forest plots were generated using the forestplot package. The nomogram plots were built employing rms package. *p*-value < 0.05 was taken as statistically significant.

## 3 Results

### 3.1 Distinct expression pattern in KIRC comparing to normal samples

**FIGURE 1:**
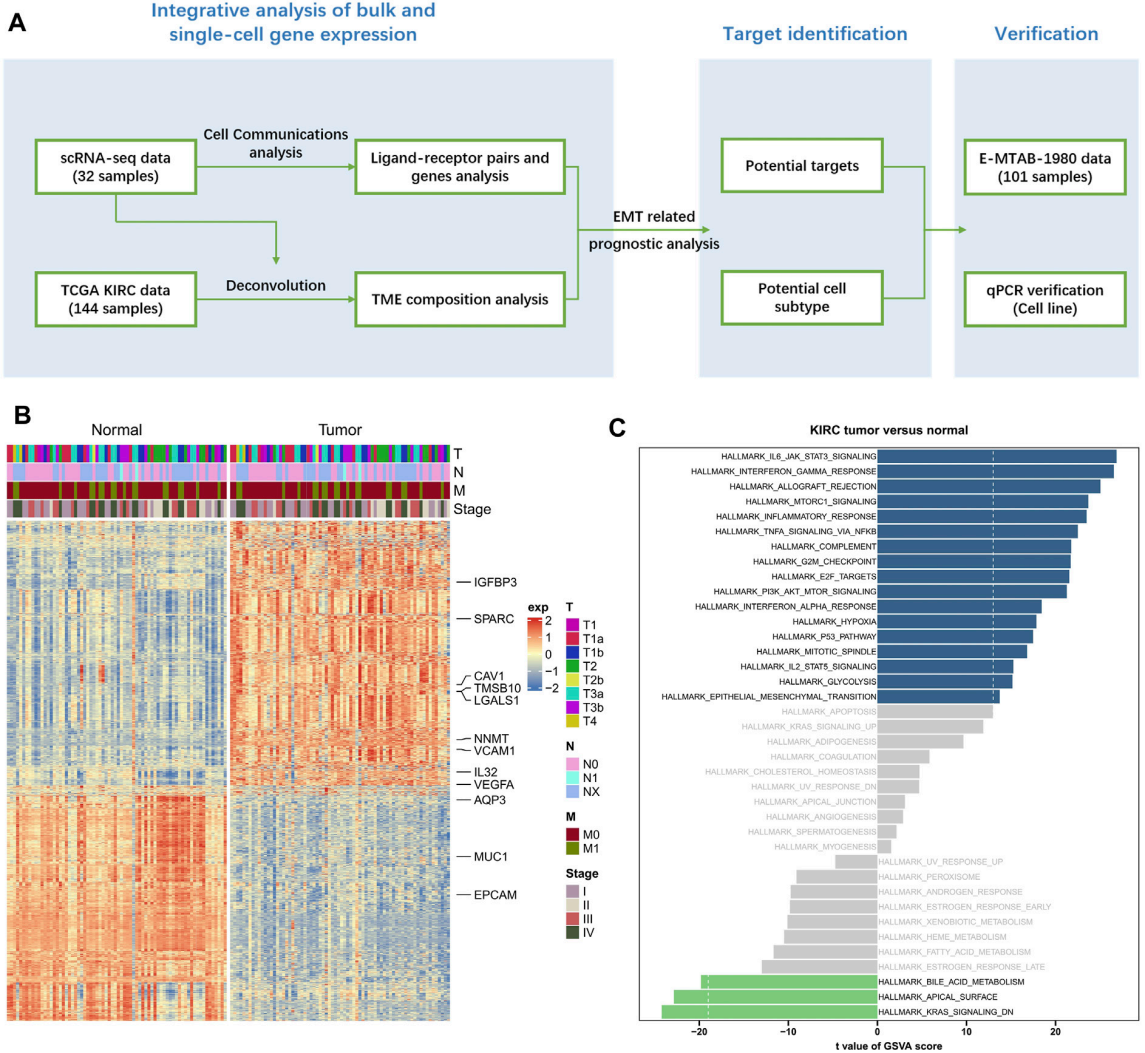
Distinct expression pattern in KIRC samples comparing to normal samples. **(A)** The overall workflow of this study. **(B)** The heat map of DEGs between KIRC samples and paired normal samples. **(C)** The GSVA results based on all 2,249 significant DEGs.

In this work, KIRC scRNA-seq data and bulk RNA-sequencing data have been jointly analyzed, and the overall workflow was displayed in Figure 1A. Totally 72 KIRC samples and the corresponding paired adjacent samples were downloaded from the TCGA-KIRC cohort, which then underwent differential gene expression analysis. Compared to adjacent samples, there were totally 2,249 significant differentially expressed genes (DEGs) in KIRC samples (|logFC| > 1.5, false discovery rates (FDR) < 0.05, *p* < 0.05), comprising 1,233 upregulated genes and 1,016 downregulated genes (Figure 1B).

The above 2,249 DEGs were subjected to a GSVA analysis, in order to obtain more functional information. We found that 1,233 upregulated genes and 1,016 downregulated genes were significantly enriched in 17 pathways and 3 pathways, respectively (*p* < 0.05, Figure 1C). Of which, we noticed that basing on upregulated genes in KIRC samples, multiple tumor related pathways have been significantly enriched, including HALLMARK Epithelial Mesenchymal Transition.

### 3.2 Significantly higher proportions of epithelial cells were found in KIRC samples at a single cell resolution

**FIGURE 2:**
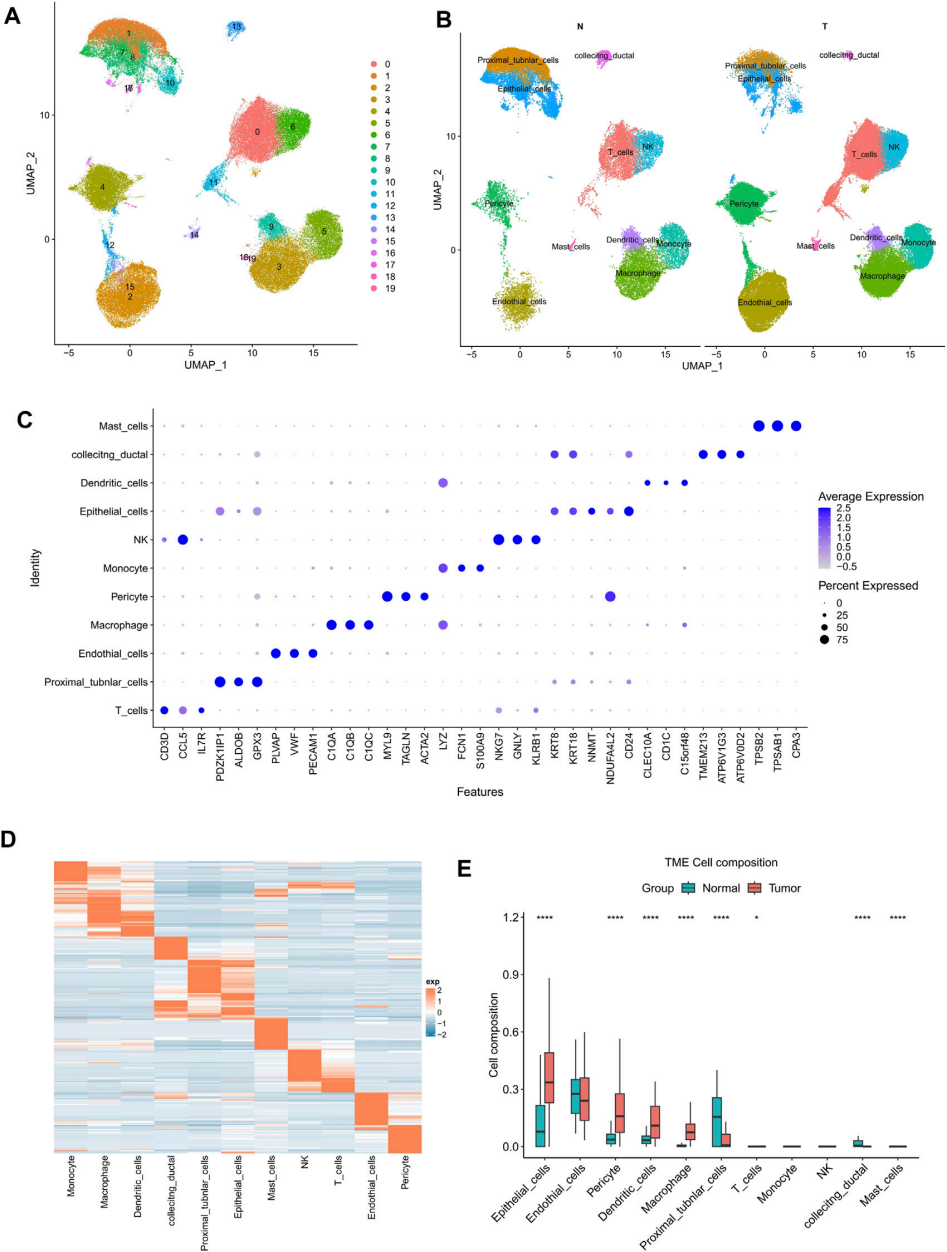
Significantly higher proportions of epithelial cells were found in KIRC samples at a single cell resolution. **(A)** Totally 76,726 cancerous tissue cells and 38,086 adjacent cells were filtered using UMAP (resolution = 0.3). **(B)** KIRC and normal samples were annotated into 11 cell types. **(C)** The dotplot showed the marker genes’ expression in 11 types of cells. **(D)** The heat map of marker genes’ expression in KIRC. **(E)** The cell composition of KIRC and normal samples. The *p*-value was determined by Wilcoxon’s rank-sum test. *p < 0.05; ****p < 0.0001.

Next, three scRNA-seq datasets GSE178481, GSE156632, GSE159115 were downloaded and analyzed. A total of 16 KIRC samples and the 16 corresponding paired normal samples were maintained for our subsequent analysis. After filtrating, 114,812 cells were obtained, including 76,726 cancerous tissue cells and 38,086 adjacent cells. UMAP analysis indicated that all cells were clustered into 20 clusters (Figure 2A). Then, all cells were annotated into 11 cell types, basing on the known marker genes (Figure 2B). Of which, CD3D, CCL5, IL7R were included as T cell markers, PDZK1IP1, ALDOB, GPX3 were included as Proximal tubule cell markers, PLVAP, VWF, PECAM1 were included as endothelial cell markers, C1QA, C1QB, C1QC were included as macrophage cell markers, MYL9, TAGLN, ACTA2 were included as pericyte cell markers, LYZ, FCN1, S100A9 were included as monocyte cell markers, NKG7, GNLY, KLRB1 were included as natural killer cell markers, KRT8, KRT18, NNMT, NDUFA4L2, CD24 were included as epithelial cell markers, CLEC10A, CD1C were included as dendritic cell markers, TMEM213, ATP6V1G3, ATP6V0D2 were included as collecting ductal cell markers, and TPSB2, TPSAB1, CPA3 were included as mast cell markers (Figure 2C).

The expression data of the above 11 cell types were analyzed employing scMappR. The signature expression matrix of all cell types was obtained (Figure 2D). Taking the signature expression matrix as the input data of CIBERSORT, the KIRC samples from TCGA cohort were then subjected a deconvolution analysis. The results indicated that epithelial cells and other 7 types of cells exhibited significantly differential cell proportions between KIRC samples and adjacent samples (Figure 2E). Besides, among all 11 cell types, significantly higher proportion of epithelial cells was observed in KIRC samples (Figure 2E). Significantly higher proportion of epithelial cells probably contributed more to the distinct expression pattern of KIRC samples.

### 3.3 Differentially expressed candidate genes in epithelial cells in KIRC samples

**FIGURE 3:**
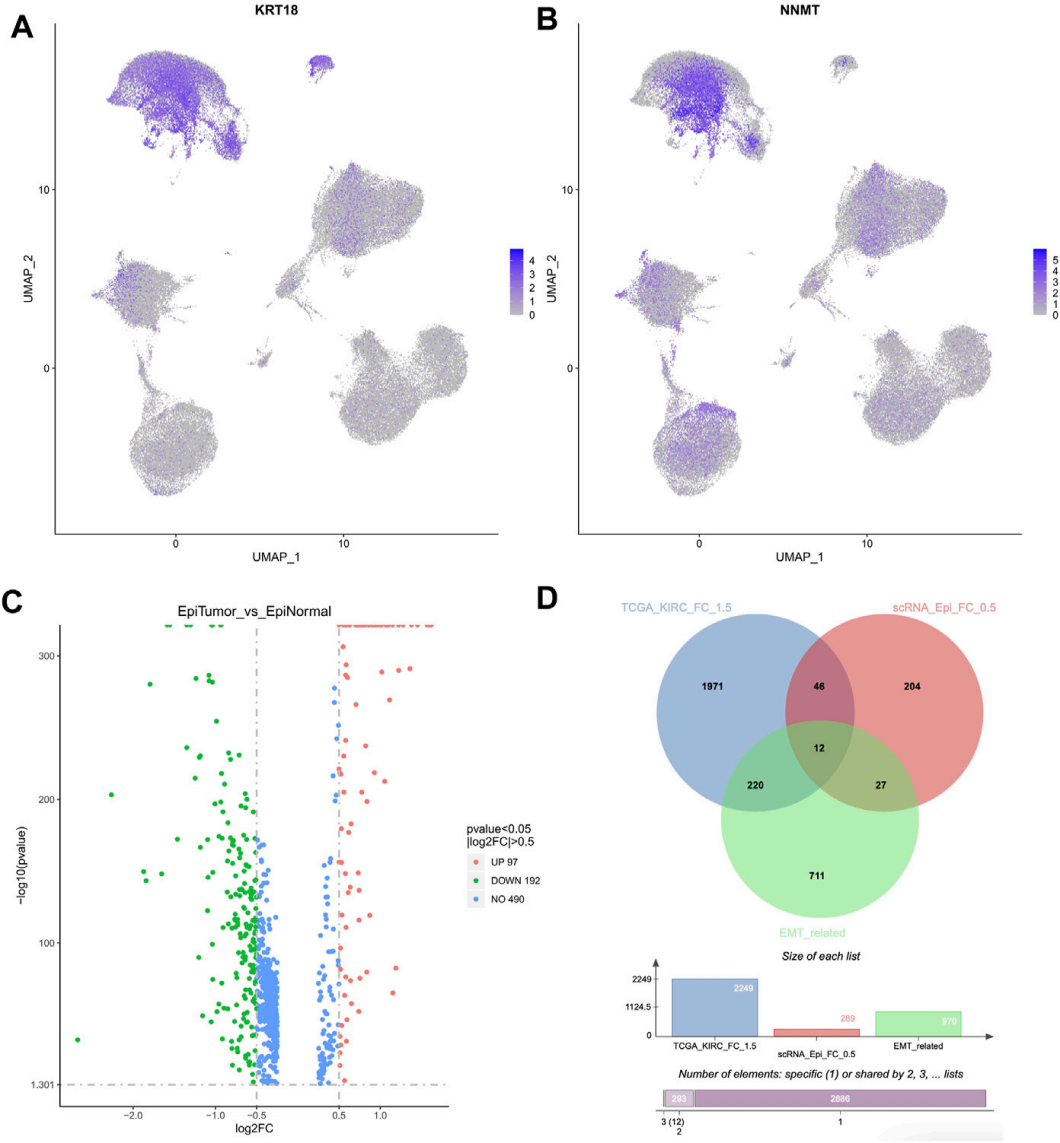
Differentially expressed candidate genes in epithelial cells in KIRC samples. **(A,B)** The feature plot indicated the KRT18 and NNMT expression in all cell types. KRT18 was the marker of epithelial cells, and NNMT was the marker of tumor cells. **(C)** The DEGs identified in epithelial cells in KIRC samples. **(D)** The Venn diagram based on 2,249 DEGs (TCGA cohort), 289 DEGs (single cell datasets), and 970 EMT related genes.

Accordingly, we have focused on the epithelial cells in KIRC samples. Based on the marker genes, the epithelial cells were found, including cluster 7, 8, 10, 16, 17 (Figures 3A, B). Compared to epithelial cells in normal cells, 289 DEGs were identified in epithelial cells in KIRC samples, of which 97 genes were upregulated and 192 were downregulated (|logFC| > 0.5, *p*-value < 0.05, Figure 3C).

Considering that the DEGs obtained based on TCGA cohort were significantly enriched in Epithelial Mesenchymal Transition pathway, 970 EMT related genes were downloaded from EMTome database (http://www.emtome.org) and CancerSEA database (http://biocc.hrbmu.edu.cn/CancerSEA/home.jsp) (Supplementary Table S6). Next, a cross analysis was performed on the above 2,249 DEGs (TCGA cohort), 289 DEGs (single cell datasets), and 970 EMT related genes. Totally 12 overlapped genes were finally identified, including IGFBP3, SPARC, CAV1, TMSB10, LGALS1, NNMT, VCAM1, IL32, VEGFA, AQP3, MUC1, EPCAM (Figure 3D). Of them, in TCGA cohort, 9 genes were significantly upregulated, and 3 genes were significantly downregulated (Figure 1B). Notably, SPARC, CAV1, TMSB10, LGALS1, and VEGFA were also significantly upregulated in epithelial cells in KIRC samples, which were regarded as the hub candidate genes in our following analysis.

### 3.4 Reliable EMT related prognostic signature was constructed for KIRC patients

**FIGURE 4:**
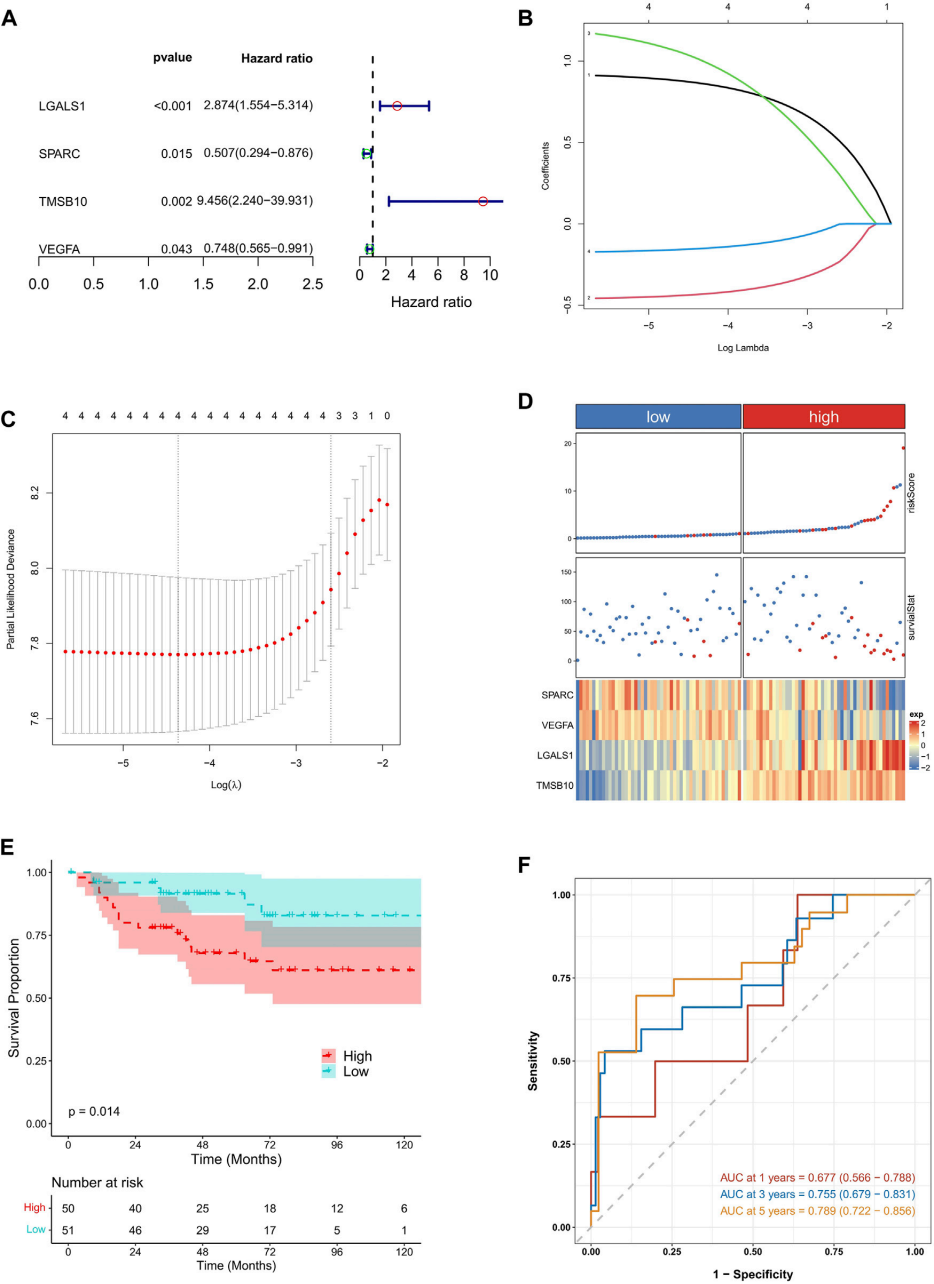
Identification of EMT related prognostic genes and prognostic signature construction in KIRC. **(A)** The results of univariate Cox regression analysis conducted on candidate EMT related genes. **(B,C)** The results of LASSO Cox regression analysis. The lambda. min value was 4. **(D)** The samples distribution based on high and low risk score. **(E)** The survival analysis between high and low risk KIRC patients. **(F)** The time dependent ROC analysis.

Subsequently, the five hub candidate genes were then subjected to an univariate Cox regression analysis to screen the KIRC prognosis related genes. We found that SPARC, TMSB10, LGALS1, and VEGFA showed significant prognostic value in KIRC (Figure 4A). To further optimize the prognostic genes, the LASSO Cox regression analysis was conducted on the above four genes, and the results indicated that there were still four genes according to lambda.min value (Figures 4B, C).

**FIGURE 5:**
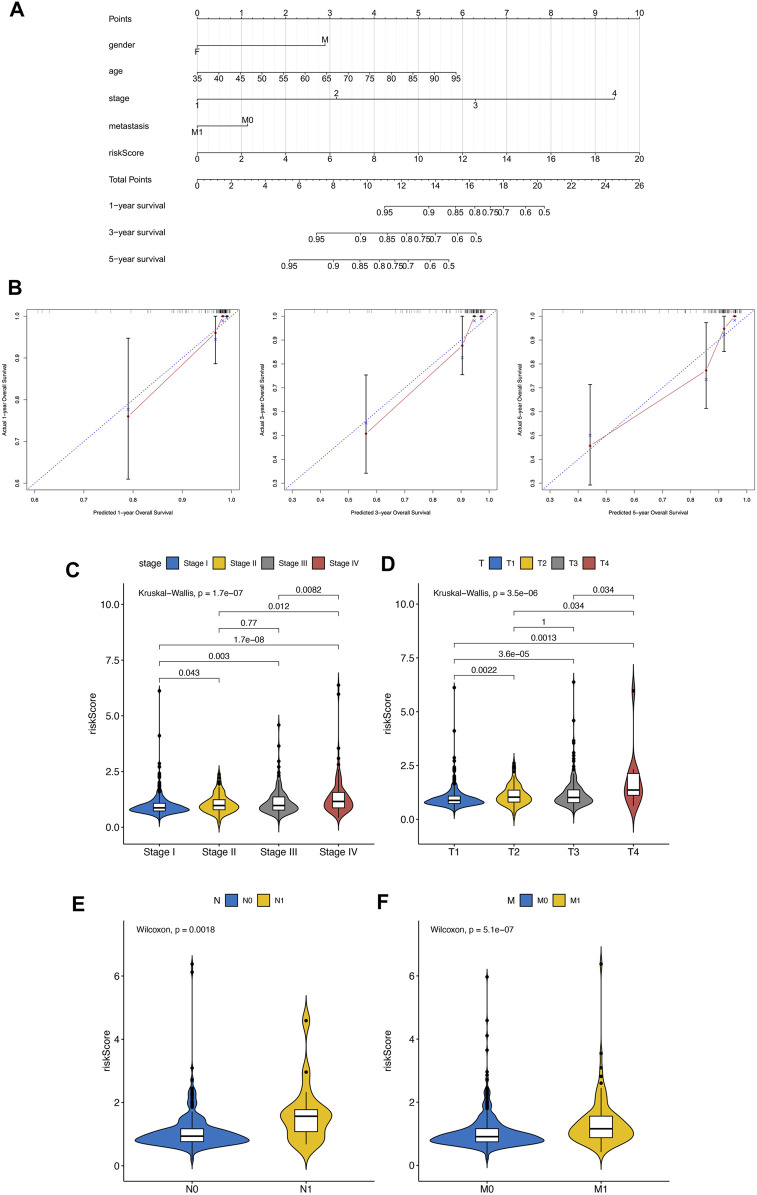
**(A–D)** Difference analysis between clinical pathological characteristics and risk score. **(E)** Nomogram model was also constructed based on risk score, gender, age, stage, and metastasis status. **(F)** The 1-, 3-, 5-year calibration curves of Nomogram.

Based on SPARC, TMSB10, LGALS1, and VEGFA, the EMT related risk score was then built. To demonstrate the performance of the model, we firstly applied it to the TCGA training cohort, which had a high accuracy in predicting of prognosis (Supplementary Figures S1A–C). Also the mutation data of TCGA-KIRC was employed to explore the relationship between risk score and TMB. The correlation between risk score and TMB was moderate and significant. Moreover, the TMB in high-risk groups was significantly higher than that in low-risk groups (Supplementary Figures S2A–D). Next the KIRC samples in E-MTAB-1980 dataset were divided into high and low risk groups, according to the median risk score. We found that high risk KIRC patients had significantly worse prognosis (*p* < 0.05) comparing to low risk patients (Figures 4D, E). Moreover, the time dependent ROC analysis suggested that the AUC values of 1-, 3-, 5-year was 0.661, 0.623, 0.613, respectively (Figure 4F), implying a good predictive performance of the EMT related risk score. Next, risk score was explored according to the related clinical pathological characteristics of KIRC samples. The results showed that the risk score has a significant difference between different pathological groups based on stage and TNM status (Figures 5A–D). Additionally, a Nomogram model was also constructed based on risk score, gender, age, stage, and metastasis status (Figure 5E). The 1-, 3-, 5-year calibration curves showed that the Nomogram model involving EMT related risk score could reliably predict KIRC patients’ prognosis (Figure 5F), which further evidenced the good predictive performance of the EMT related risk score.

### 3.5 Distinct drug sensitivity between risk groups

The distinct immunotherapy response between the high- and low-risk groups were investigated using the R package OncoPredict. Among the 198 chemotherapeutic drugs, Dactinomycin_1911, Elephantin_1835 and ERK_6604_1714 had significantly lower predicted IC50 values in the higher than in the lower risk group with the top 3 negative correlation between IC50 and risk score (Supplementary Figures S3A–F).

### 3.6 Significantly enhanced epithelial cell cell-cell communications were observed in VEGF signaling pathway

**FIGURE 6:**
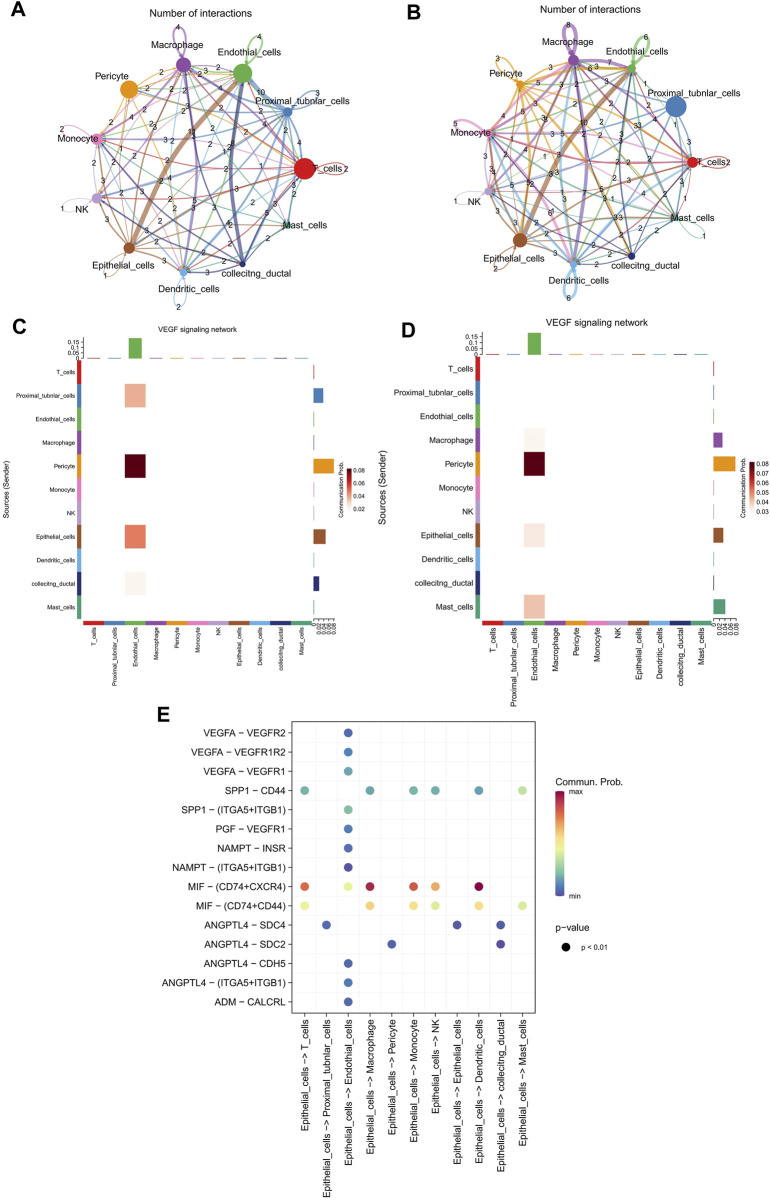
Significantly enhanced epithelial cell cell-cell communications were observed in VEGF signaling pathway. **(A,B)** The possible cell-cell interaction numbers in KIRC cells and adjacent cells, separately. **(C,D)** The cell communication of 11 cell types in VEGF pathway network in KIRC cells and adjacent cells, respectively. **(E)** Potential ligand-receptor interactions in KIRC cells.

Furthermore, to obtain more cell talk details between KIRC and adjacent samples at a single cell resolution, the cell-cell communication analysis was conducted on KIRC cells and adjacent cells using Cellchat. The possible cell-cell interaction numbers in KIRC cells and adjacent cells were displayed in Figures 6A, B, respectively.

As a famous process, EMT has been indicated to confer efficient tumorigenicity to tumor cells via activating vascular endothelial growth factor (VEGF) pathway (Fantozzi et al., 2014). Thus, we have also analyzed the cell communication of 11 cell types in VEGF pathway network, in order to better understand the EMT related functions. Our results suggested that epithelial cells and endothelial cells showed higher communication probability in KIRC samples than in adjacent samples (Figures 6C, D). The communication probability of epithelial cells in KIRC samples and adjacent samples were 0.21 and 0.14, separately. Moreover, the potential ligand-receptor interactions in KIRC cells were predicted, and there were more ligand-receptor interactions between epithelial cells and endothelial cells (Figure 6E). Moreover, VEGFA, as one of the four genes in the prognostic signature, is a member of the PDGF/VEGF growth factor family. The Kaplan-Meier survival curve predicted that VEGFA could be a factor for patients’ prognosis (Supplementary Figures S4A, B).

### 3.7 EMT related hub genes’ validation and functions *in vitro*

**FIGURE 7:**
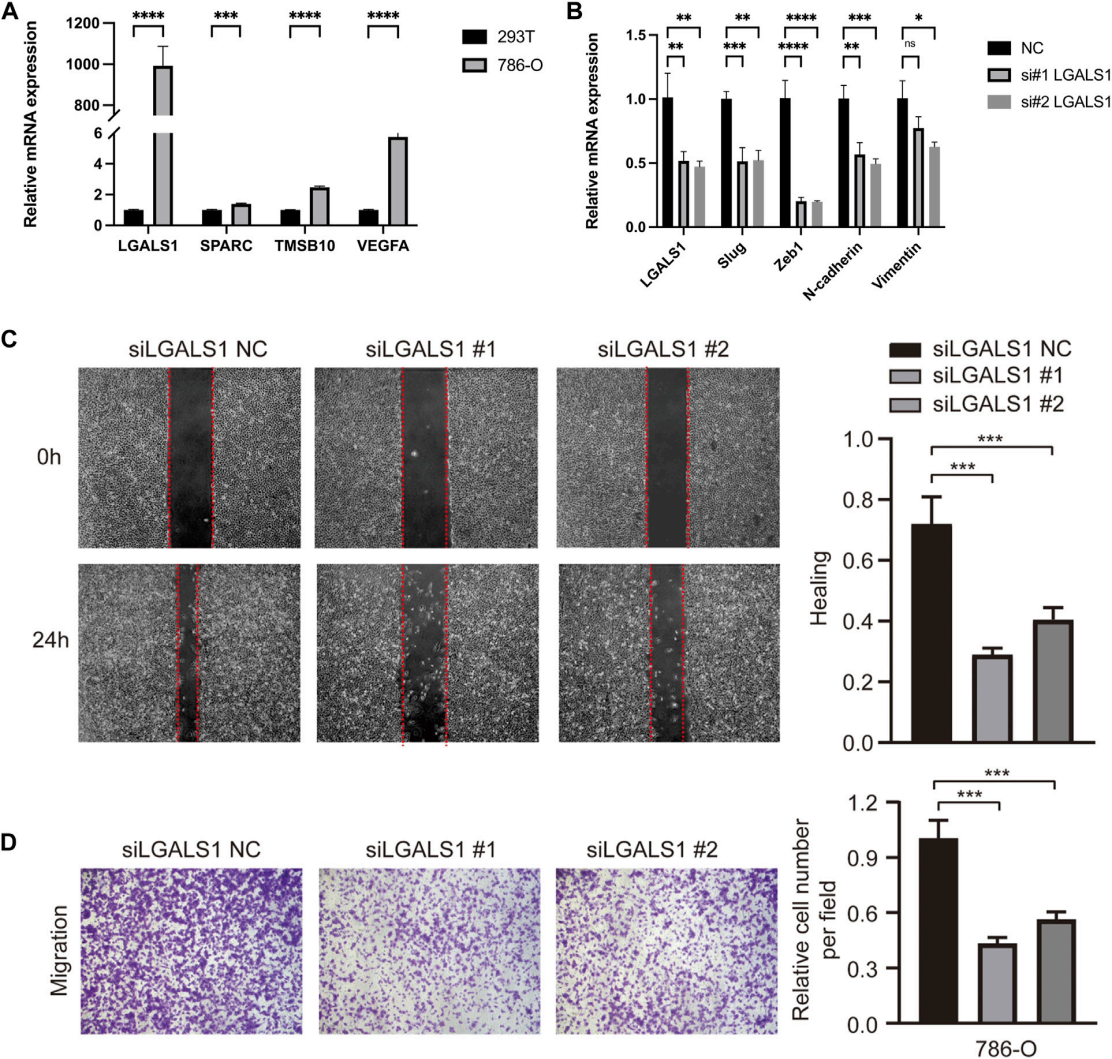
EMT related hub genes’ validation and functions *in vitro*. **(A)** The relative mRNA expressions of SPARC, TMSB10, LGALS1, and VEGFA in cell lines, detected by q-PCR. **(B)** EMT related markers in LGALS1 silencing 786-O cells. **(C)** The scratch assay in LGALS1 silencing 786-O cells. **(D)** The transwell migration and invasion assays in LGALS1 silencing 786-O cells. Three independent experiments were performed in 786-O cell lines and all data were presented as the means ± SD of three independent experiments. *p < 0.05, **p < 0.01, ***p < 0.001, ****p < 0.0001.

Furthermore, the expressions of hub EMT related genes in risk score, SPARC, TMSB10, LGALS1, and VEGFA, were validated *in vitro*. The results of qRT-PCR showed that there were significantly higher mRNA expressions of SPARC, TMSB10, LGALS1, and VEGFA in 786-O cells, comparing to 293T cells (Figure 7A).

Of which, we noticed that LGALS1 showed the most significant expression difference between tumor and normal cells, thus effects of LGALS1 on KIRC cell migration and invasion ability were further explored. Then, the interference RNA and control were transfected into 786-O cells, and the silencing of LGALS1 was confirmed by RT-qPCR in 786-O cells (Figure 7B). Moreover, knockdown of LGALS1 significantly downregulated the mRNA expressions of multiple EMT related markers, including Slug, Zeb1, N-cadherin, and Vimentin (Figure 7B), implying LGALS1 probably contributed to suppress EMT process. The scratch assay indicated that si-LGALS1 significantly suppressed the 786-O cell migration capacity compared to control cells (Figure 7C). Besides, results of transwell migration and invasion assays suggested that migration and invasion ability of KIRC cells were significantly inhibited by si-LGALS1 (Figure 7D). Collectively, our data indicated that si-LGALS1 could inhibit the cell migration and invasion ability of KIRC, which might be involved in suppressing EMT process.

## 4 Discussion

Despite great efforts have been devoted into the therapeutic development and survival improvement, KIRC is prone to metastasize and the 5-year overall survival of metastatic KIRC patients is still frustrated (Yang et al., 2022). Obviously, it is quite imperative for improving prognosis to mine more details of pathogenesis of KIRC, especially regarding the metastasis of KIRC patients. In our present study, we have not only intensively integrated the KIRC scRNA-seq data and bulk RNA-sequencing data, but also focused on the EMT process and its related genes in KIRC. Finally, hub genes SPARC, CAV1, TMSB10, LGALS1 were identified in epithelial cells in KIRC, based on which, a reliable EMT related prognostic signature was constructed for KIRC patients, exhibiting excellent prognostic value. More importantly, we found that si-LGALS1 could inhibit the cell migration and invasion ability of KIRC, which might be involved in suppressing EMT process *in vitro*.

Basing on bulk RNA-sequencing data in TCGA-KIRC cohort, we totally identified 2,249 significant DEGs in KIRC samples, meanwhile a significant distinct gene expression pattern was indeed found in KIRC samples comparing to normal samples. Hence, the functional information of the DEGs was then analyzed, of which Epithelial Mesenchymal Transition pathway attracted our attention. EMT often exerted tumor promoting role in many cancers. Typically, EMT occurred along with downregulated epithelial markers (like E-cadherin) and upregulated mesenchymal markers (like Vimentin and N-cadherin), leading to the detachment and elongation of the cells (Zhu et al., 2013). It has been suggested to play crucial roles in initial invasion and metastasis cascades of tumor cells, including in KIRC (Das et al., 2019; Gao et al., 2022). It follows that there is complicated potential association between the distinct expression pattern in KIRC and EMT.

Accordingly, to further clarify the potential crucial contributors of the distinct gene expression pattern in KIRC at a single cell resolution, scRNA-seq datasets and signature matrix were employed to characterize the important cell composition in KIRC. Our data indicated that among all 11 cell types in KIRC, significantly higher proportion of epithelial cells was observed. Next, focusing on the epithelial cells in KIRC, 289 DEGs were identified in epithelial cells in KIRC. These data based on scRNA-seq data could just be connected with our findings in bulk RNA-sequencing data. Although we cannot curtly conclude whether significantly higher epithelial cell proportions in KIRC would refer to a higher probability of EMT onset, our results still implied a promising probability involving epithelial cells and EMT, KIRC metastasis. In the dynamic and reversible transition during EMT, the typical features of epithelial cells would be deprived, like junctions and baso-apical polarity, whereas they would gradually be acquired back-to-front polarity and the ability to migrate and invade surrounding tissues (Manfioletti and Fedele, 2022). It has been demonstrated that the high plasticity of EMT is mainly regulated by the epigenetics modifications, predominantly involving histone modification of core EMT related transcription factors, for instance SNAIL, SLUG, TWIST, and ZEB (Manfioletti and Fedele, 2022). In KIRC, many studies have evidenced that the EMT was triggered by the regulatory axis including the above transcription factors, which then contributed the carcinogenesis features of tumor (Liu et al., 2018; Liu et al., 2021b).

Subsequently, the cross analysis of all DEGs and 970 EMT related genes obtained 12 overlapped genes, of which SPARC, CAV1, TMSB10, LGALS1, and VEGFA were significantly upregulated in epithelial cells and KIRC. After univariate Cox regression analysis and LASSO Cox regression analysis, we finally identified four hub EMT related genes in KIRC, including SPARC, TMSB10, LGALS1, and VEGFA. The EMT related prognostic signature based on the above four hub genes exhibited excellent prognostic value in KIRC. SPARC (Secreted protein, acidic and rich in cysteine) encodes a small molecule glycoprotein osteopontin, which mainly involves in the regulation of cell adhesion, proliferation, and spreading (Schellings et al., 2009). As an EMT related gene, the prognostic value of SPARC has been reported in many tumors, like breast cancer (Schellings et al., 2009) and lung cancer (Ma et al., 2022). Meanwhile, SPARC has also been studied solely in KIRC, which implied that it might be a key mediator of TGF-β-induced renal cancer metastasis (Bao et al., 2021). Their work could partly explain the prognostic role of SPARC we found. TMSB10 (Thymosin β10), as a member in β-thymosin family, participates in the control of the cytoskeletal microfilament system as actin-binding protein (Bao et al., 2021). Recently, its role in tumor development has been widely studied. For instance, declined TMSB10 expression was involved in the DNMT1/miR-152-3p/TMSB10 axis to suppress the colorectal cancer development (Wang et al., 2020). High expression of TMSB10 was associated with the bladder cancer progression and poor prognosis of patients (Wang et al., 2019). Additionally, TMSB10 has been indicated to be overexpressed in RCC, promoting the tumor cell malignant metastasis by inducing EMT (Xiao et al., 2019), which was consistent with our data. Moreover, LGALS1 (Galectin-1) could interact with extracellular matrix glycoproteins, exerting crucial roles in cell division, migration, adhesion, and invasion (Li et al., 2023a). Considering its most significant aberrant expression *in vitro*, we further focused on its functions and finally found that si-LGALS1 could inhibit the cell migration and invasion ability of KIRC, which might be involved in suppressing EMT process. Numerous studies have provided the consistent findings that it functioned as a tumor promoting factor in multiple cancers, such as in gastric cancer (Zhang et al., 2022) and ovarian cancer (Li et al., 2023b). Collectively, all above evidences have directly or indirectly supported our findings, providing potential clues regarding the prognostic value of our EMT related signature of KIRC.

As for VEGFA, it is an important mediator of vascular development such as angiogenesis, meanwhile angiogenesis is a pivotal event in tumor progression (Zhang et al., 2023). In our study, significantly enhanced epithelial cell cell-cell communications were observed in VEGF signaling pathway in KIRC. KIRC has been widely known as a tumor with rich angiogenesis, meanwhile angiogenesis is a necessary process of tumor development (Li D. X. et al., 2023). It has been indicated that VEGF could enhance the angiogenesis and cell proliferation of endothelial cells in KIRC (Edeline et al., 2012). Therefore, the VEGF related signal was also a significant aspect in the distinct expression pattern formation of KIRC, which deserved to be further investigated in our future work.

## 5 Conclusion

In conclusion, we have constructed a novel powerful EMT related prognostic signature for KIRC patients, based on SPARC, TMSB10, LGALS1, and VEGFA. Of which, si-LGALS1 could inhibit the cell migration and invasion ability of KIRC, which might be involved in suppressing EMT process *in vitro*. Furthermore, combining KIRC scRNA-seq data and bulk RNA-sequencing data mining, a significant distinct expression pattern was observed in KIRC comparing to normal samples, involving in the EMT related signals. Our data are expected to uncover more details associating with EMT in KIRC, meanwhile the EMT related prognostic signature is conducive to developing better clinical management strategy for KIRC patients.

## Data Availability

The original contributions presented in the study are included in the article/Supplementary Material, further inquiries can be directed to the corresponding author.

## Supplementary material

SUPPLEMENTARY FIGURE S1
**(A)** The samples distribution based on high and low risk score. **(B)** The survival analysis between high and low risk TCGA samples. **(C)** The time dependent ROC analysis.

SUPPLEMENTARY FIGURE S2
**(A)** Overall description of the mutational landscape in the high risk groups. **(B)** Overall description of the mutational landscape in the low risk groups. **(C)** Difference analysis of TMB between the high and low risk groups. **(D)** Correlation analysis of TMB and risk score of patients.

SUPPLEMENTARY FIGURE S3
**(A–C)** Difference analysis of IC50 between high- and low-risk groups. **(D–F)** Correlation analysis of IC50 between high- and low-risk groups.

SUPPLEMENTARY FIGURE S4
**(A)** ROC analysis in TCGA cohort. **(B)** ROC analysis in E-MTAB-1980 cohort.
