# Supplementary figures and images for: Construction of EMT related prognostic signature for kidney renal clear cell carcinoma, through integrating bulk and single-cell gene expression profiles
Source: Front Pharmacol. 2023 Nov 15;14:1302142. doi: 10.3389/fphar.2023.1302142 (PMC10684753; doi:10.3389/fphar.2023.1302142)

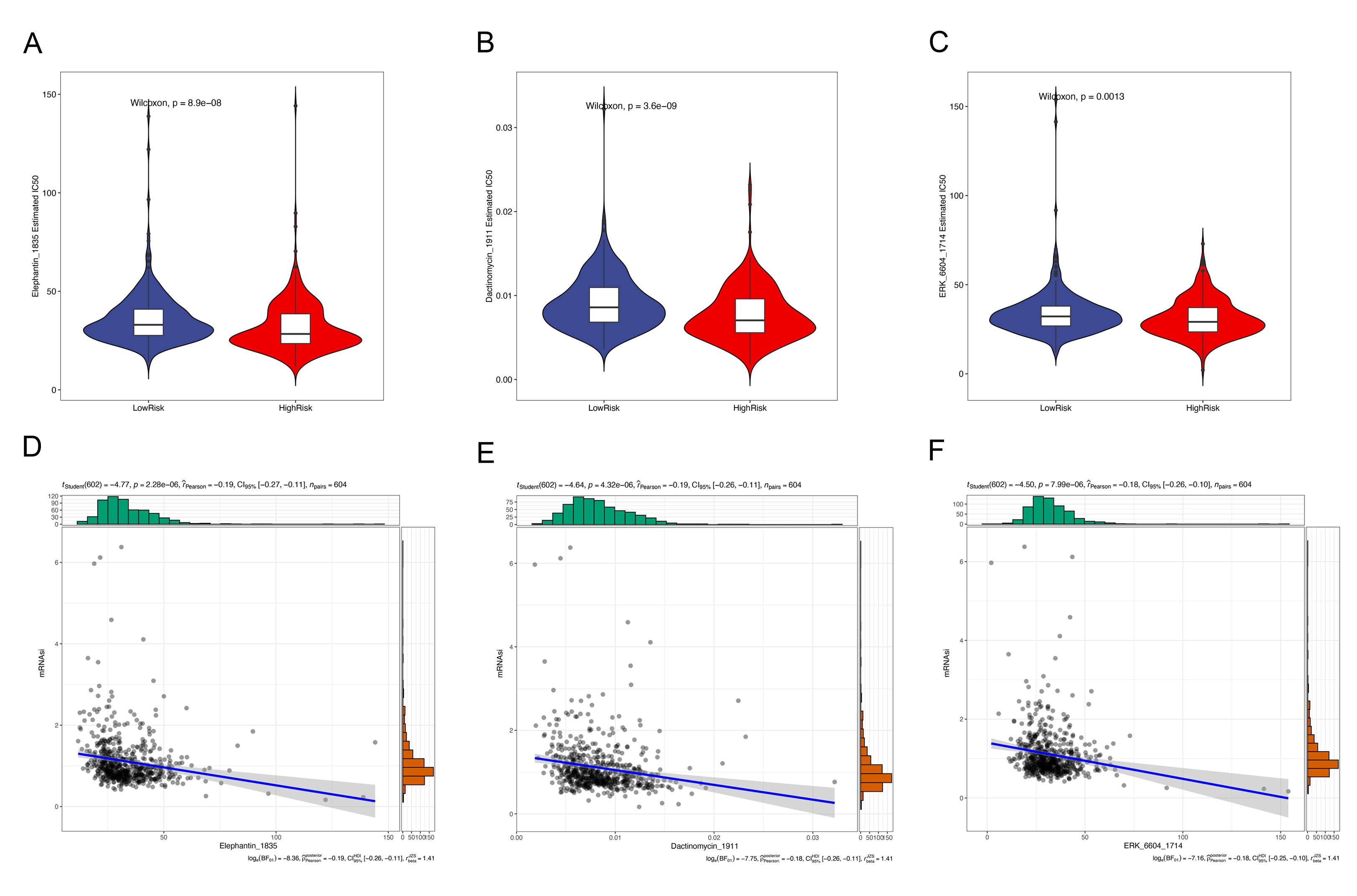

Supplement: Supplementary file 3 [file Image3.JPEG]

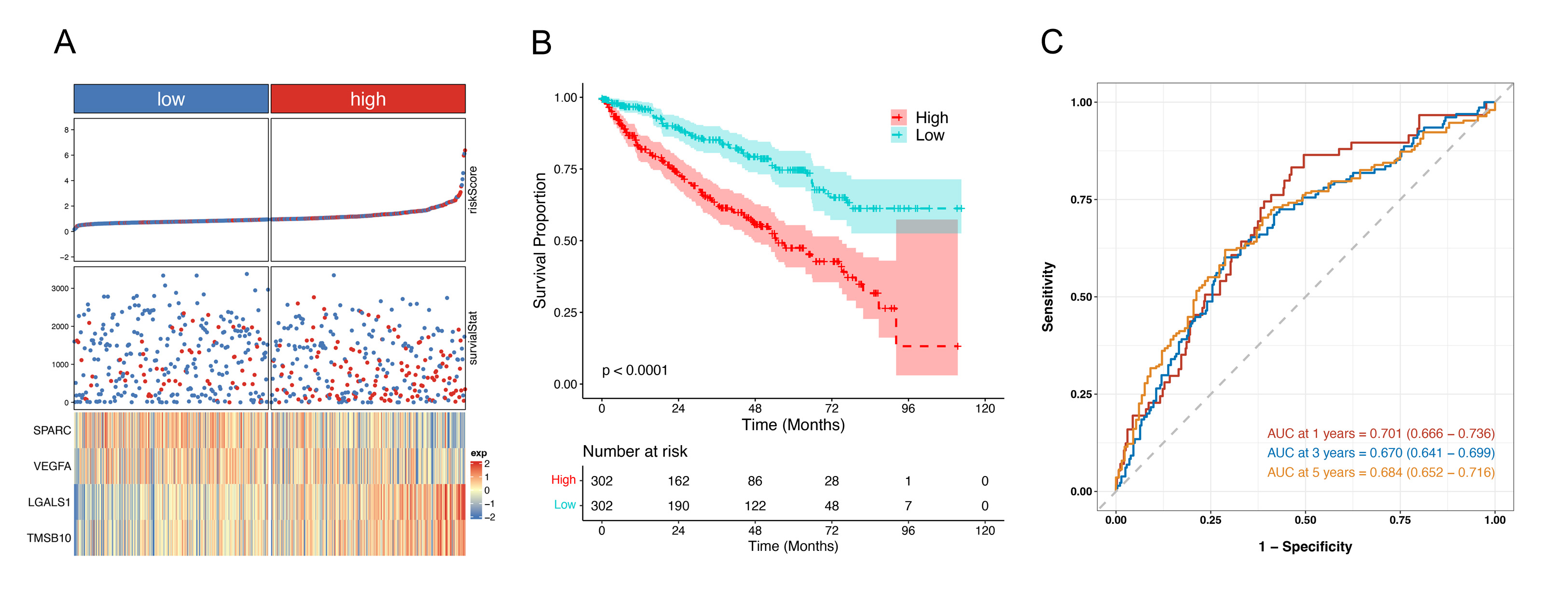

Supplement: Supplementary file 6 [file Image1.JPEG]

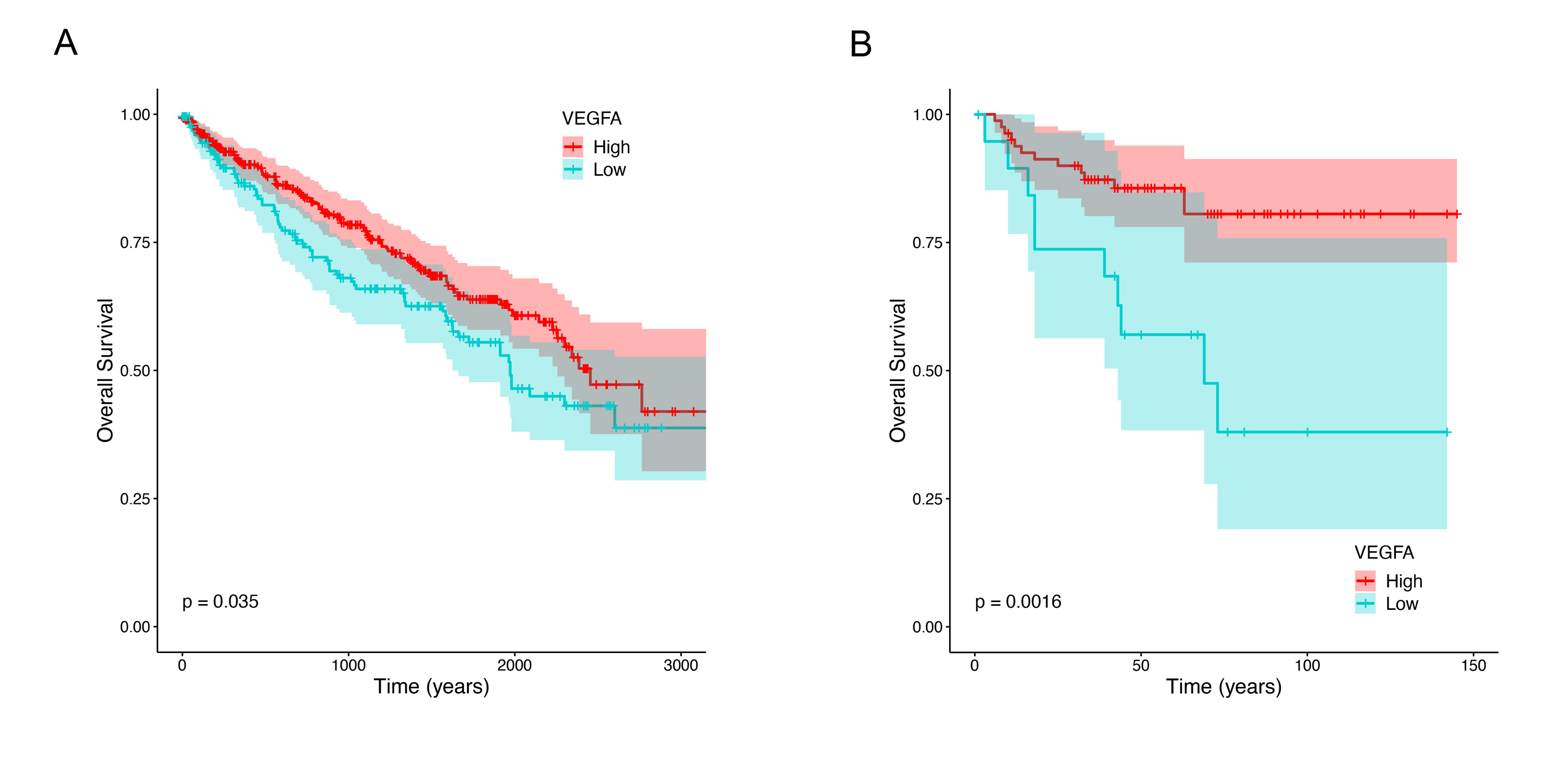

Supplement: Supplementary file 7 [file Image4.JPEG]

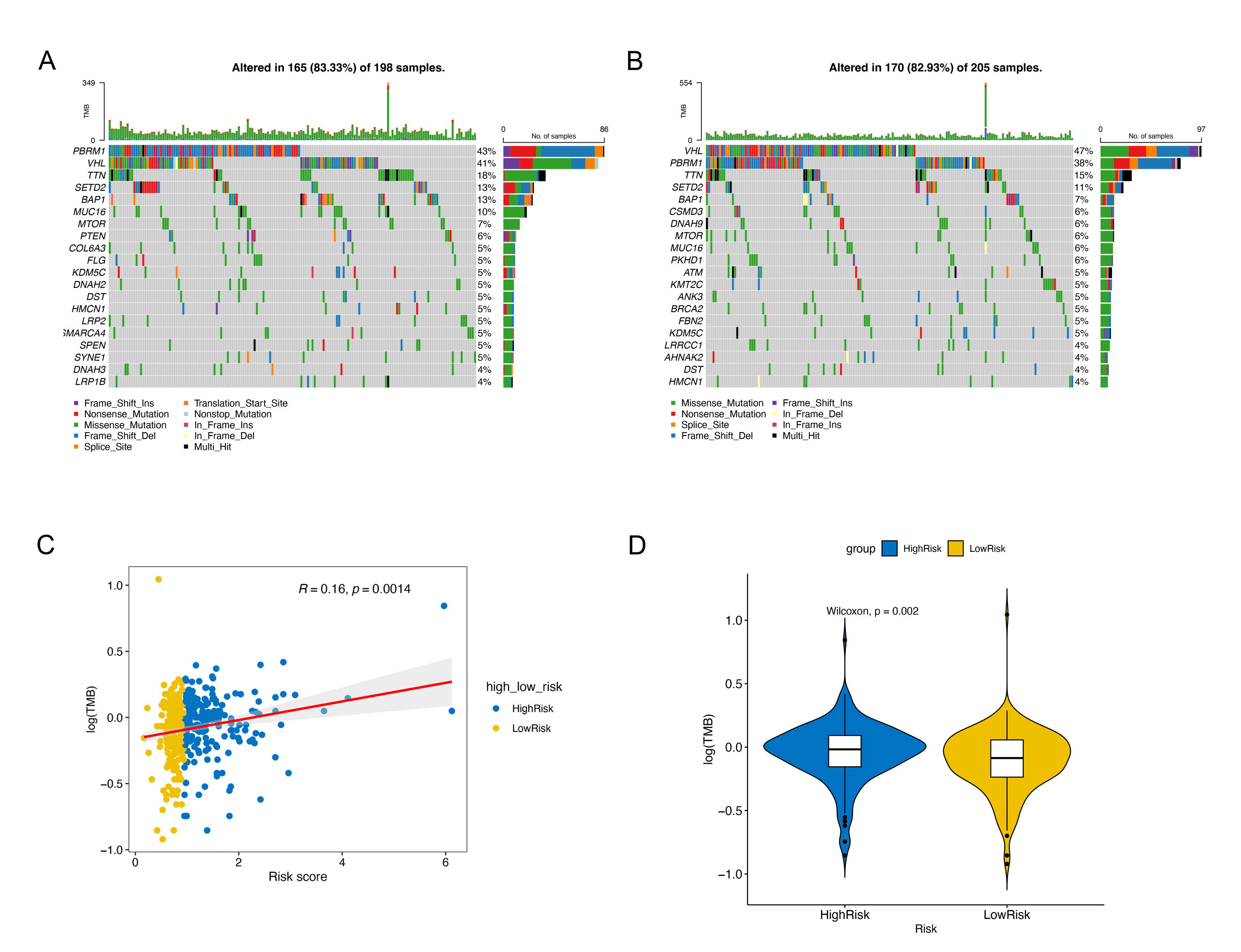

Supplement: Supplementary file 8 [file Image2.JPEG]
